# Machine Learning-Based Prediction of Complications and Prolonged Hospitalization with the GLIM Criteria Combinations Containing Calf Circumference in Elderly Asian Patients

**DOI:** 10.3390/nu15194146

**Published:** 2023-09-26

**Authors:** Shan-Shan Ren, Kai-Wen Zhang, Bo-Wen Chen, Chun Yang, Rong Xiao, Peng-Gao Li, Ming-Wei Zhu

**Affiliations:** 1 Department of Clinical Nutrition, Beijing Hospital, National Center of Gerontology, Institute of Geriatric Medicine, Chinese Academy of Medical Sciences, Beijing 100730, China; renshanshan4288@bjhmoh.cn; 2 The Key Laboratory of Geriatrics, National Center of Gerontology, National Health Commission, Beijing Hospital, Institute of Geriatrics, Institute of Geriatric Medicine, Chinese Academy of Medical Sciences, Beijing 100730, China; 3 School of Public Health, Capital Medical University, Beijing 100069, China; zhangkaiwen@ccmu.edu.cn (K.-W.Z.); yangchun@ccmu.edu.cn (C.Y.);; 4 Beijing Key Laboratory of Environmental Toxicology, Beijing 100069, China; 5 Beijing Key Laboratory of Clinical Epidemiology, Beijing 100069, China; 6 Sir Run Run Shaw Hospital, Hangzhou 310016, China; chenbowen@ccmu.edu.cn

**Keywords:** calf circumference, malnutrition, GLIM, in-hospital complications, prolonged length of hospital stay, elderly

## Abstract

Background and aims: Malnutrition is widely present and influences the prognosis of elderly inpatients, so it is helpful to be able to identify it with a convenient method. However, in the widely accepted criteria for malnutrition, the Global Leadership Initiative on Malnutrition (GLIM), a lot of metrics can be used to define the phenotypic and etiological criteria. To identify muscle mass reduction, anthropometric parameters such as calf circumference (CC) and hand grip strength (HGS) are preferable to other expensive methods in many situations because they are easy and inexpensive to measure, but their applicability needs to be verified in specific clinical scenarios. This study aims to verify the value of CC- and HGS-identified muscle loss in diagnosing malnutrition and predicting in-hospital complications (IHC) and prolonged length of hospital stay (PLOS) in elderly inpatients using machine learning methods. Methods: A sample of 7122 elderly inpatients who were enrolled in a previous multicenter cohort study in China were screened for eligibility for the current study and were then retrospectively diagnosed for malnutrition using 33 GLIM criteria that differ in their combinations of phenotypic and etiological criteria, in which CC or CC+HGS were used to identify muscle mass reduction. The diagnostic consistency with the subjective global assessment (SGA) criteria at admission was evaluated according to Kappa coefficients. The association and the predictive value of the GLIM-defined malnutrition with 30-day IHC and PLOS were evaluated with logistic regression and randomized forest models. Results: In total, 2526 inpatients (average age 74.63 ± 7.12 years) were enrolled in the current study. The prevalence of malnutrition identified by the 33 criteria combinations ranged from 3.3% to 27.2%. The main IHCs was infectious complications (2.5%). The Kappa coefficients ranged from 0.130 to 0.866. Logistic regression revealed that malnutrition was identified by 31 GLIM criteria combinations that were significantly associated with 30-day IHC, and 22 were significantly associated with PLOS. Random forest prediction revealed that GLIM 15 (unconscious weight loss + muscle mass reduction, combined with disease burden/inflammation) performs best in predicting IHC; GLIM 30 (unconscious weight loss + muscle mass reduction + BMI reduction, combined with disease burden/inflammation) performs best in predicting PLOS. Importantly, CC alone performs better than CC+HGS in the criteria combinations for predicting adverse clinical outcomes. Conclusion: Muscle mass reduction defined by a reduced CC performs well in the GLIM criteria combinations for diagnosing malnutrition and predicting IHC and PLOS in elderly Asian inpatients. The applicability of other anthropometric parameters in these applications needs to be further explored.

## 1. Introduction

Malnutrition is very common in elderly inpatients, which increases the risk of various complications and mortality, prolongs the length of hospital stay, and increases related costs [1]. Fortunately, malnutrition is reversible, so early and accurate identification of malnutrition is crucial for preventing adverse clinical outcomes in elderly inpatients.

The subjective global assessment (SGA) is now considered the gold standard for malnutrition diagnosis [2]. However, the implementation of SGA is constrained by its cumbersome operations that involve the assessment of a wide range of characteristics of malnutrition [3].

In 2018, several major clinical nutrition societies around the world proposed the Global Leadership Initiative on Malnutrition (GLIM) framework to simplify and standardize the diagnosis of malnutrition, which consists of three phenotypic criteria (body mass index, weight loss, and muscle mass reduction) and two etiological criteria (food intake reduction, disease or inflammation burden) [2]. As long as any one of the phenotypic criteria plus any one of the etiological criteria are met, malnutrition can be diagnosed. However, the problem is that, in different clinical settings, using more than one phenotypic and etiological criterion may be more suitable for prognosis purposes, which gives rise to a complex permutation of the three phenotypic and two etiological criteria. In different scenarios, it can be the combination of one, two, or three phenotypic criteria and one or two etiological criteria. It can be applied to patients of different ages, gender, and diseases, and used to predict different clinical outcomes (e.g., mortality, complications, prolonged length of hospital stay (PLOS), etc.). To date, many researchers have verified the application of different GLIM criteria combinations in different populations and found that the prevalence of malnutrition identified by different GLIM criteria combinations varied greatly, and its correlation with prognosis also varied greatly [4,5,6].

Moreover, there are a lot of alternative metrics that can be chosen for measuring either the phenotypic or etiological criteria. For instance, there are a lot of methods for the evaluation of muscle mass reduction, a very important phenotypic criterion for sarcopenia and malnutrition [7,8,9]. Human body composition measured with computed tomography (CT), magnetic resonance imaging (MRI), dual-energy X-ray absorption (DXA), and bioelectrical impedance analysis (BIA) can accurately describe the body composition including the skeletal muscle volume, but these methods need expensive instruments and are difficult to operate for many clinics in undeveloped regions, restricting their wide application. In contrast, anthropometric parameters such as calf circumference (CC), hand-grip strength (HGS), and mid-upper-arm circumference (MAC) are easy to measure and do not require expensive instruments, sites, or complex operations. These metrics can be efficiently measured in a very short time after simple training. The problems of anthropometric parameters include the lack of widely applicable thresholds for diagnosis because they are affected by the age, gender, race, and pathophysiological status of the subjects [10,11,12]. The applicability of these metrics in the GLIM phenotypic criteria must be verified in specific populations and clinical scenarios.

Thus, the GLIM committee encourages the validation of different GLIM criteria combinations in various clinical practices to identify the most relevant diagnostic criteria combinations for predicting specific adverse clinical outcomes such as in-hospital complications (IHCs) or prolonged length of hospital stay (PLOS) [13]. The European Society for Clinical Nutrition and Metabolism (ESPEN), the European Union Geriatric Medicine Society (EuGMS), and the GLIM committee all call for finding practical and verifiable methods to solve the gap between research and clinical practice [13,14].

Therefore, in this work, 33 combinations of the GLIM phenotypic and etiological criteria were used for malnutrition diagnosis, and their usefulness in predicting 30-day IHC (including infections, anastomotic leakage, anemia, electrolyte disorder, myocardial infarction) and PLOS (of more than 14 days) in elderly inpatients was evaluated with machine learning methods. More importantly, in these combinatory GLIM criteria, anthropometric metrics, including CC and CC+HGS were used as the main criteria for assessing muscle mass reduction. Both logistic regression and random forest models are employed to evaluate the association and performance of the GLIM-identified malnutrition in predicting 30-day IHC and PLOS in the subjects.

## 2. Materials and Methods

### 2.1. Population

The subjects were screened from a dataset of 7122 subjects who were enrolled from June to September 2014 in a large-scale prospective observational cohort study that covered 34 level-A tertiary hospitals, the highest level for general hospitals, in 18 cities in China [15,16]. All patients had a complete medical record track and a subjective global assessment (SGA) at admission. Their data were retrospectively analyzed and diagnosed for malnutrition with the different combinations of the GLIM criteria. Patients in the internal medical or surgical wards who were 65 years old or above, self-conscious, and had signed a written informed consent form were included in the present study. Patients who had been in the wards for <7 days or >30 days, or had incomplete data, were excluded from the dataset. The study protocol was approved by the ethics committee of the Beijing Hospital (No. 2014BJYYEC-022-02) and was conducted following the Declaration of Helsinki, and was registered in the China Clinical Trial Registration Center (Registration No. ChiCTR-EPC14005253).

### 2.2. Data Collection

A standardized research protocol was adopted in the present study. The data collected in this study include (1) demographic parameters: gender, age, marital status, and educational level; (2) reasons for hospitalization, medical history, weight loss, and food intake; and (3) anthropometric parameters: height, weight, MAC, CC, and HGS as measured by standard methods. All investigators received prior standardized training on obtaining the anthropomorphic measurements. CC was measured when the subjects bent their knees 90° in a sitting position, putting their feet flat on the ground. The thickest part of the lower leg was measured with an inelastic tape measure. HGS was measured with an electronic grip strength meter (EH101, Xiangshan, Guangdong, China). The subjects were seated, with shoulders adducted and elbows bent 90°. The left and right hands were measured 3 times each, taking the maximum value, with the measurement value accurate to 0.1 kg. MAC was measured when the subjects sat with their forearm on a plane and bent their elbows 90°, and the circumference of the midpoint of the upper arm was measured with an inelastic tape. (4) Laboratory parameters: whole blood cell counts and blood biochemistries including total protein, albumin, pre-albumin, triglyceride, and cholesterol.

All subjects had completed the nutritional risk screening-2002 (NRS-2002) scale [17] within 24 h of admission and had received a diagnosis of malnutrition with the SGA scale [15,18] by a trained clinician. Then, different combinations of the GLIM criteria were applied retrospectively to these subjects for the diagnosis of malnutrition.

### 2.3. Adverse Clinical Outcomes

The primary outcomes of the present study include the occurrence of various complications within 30 days of admission and a PLOS of more than 14 days. Complications are defined as any deviation from the ideal treatment process, such as infections, anastomotic leakage, anemia, electrolyte disorder, myocardial infarction, etc., except for untreated primary diseases.

### 2.4. Diagnostic Criteria for Malnutrition

#### 2.4.1. The SGA Criteria

All subjects in the present study were assessed according to the SGA criteria [15] within 24 h of hospital admission by a trained physician.

#### 2.4.2. The GLIM Criteria

**Figure 1:**
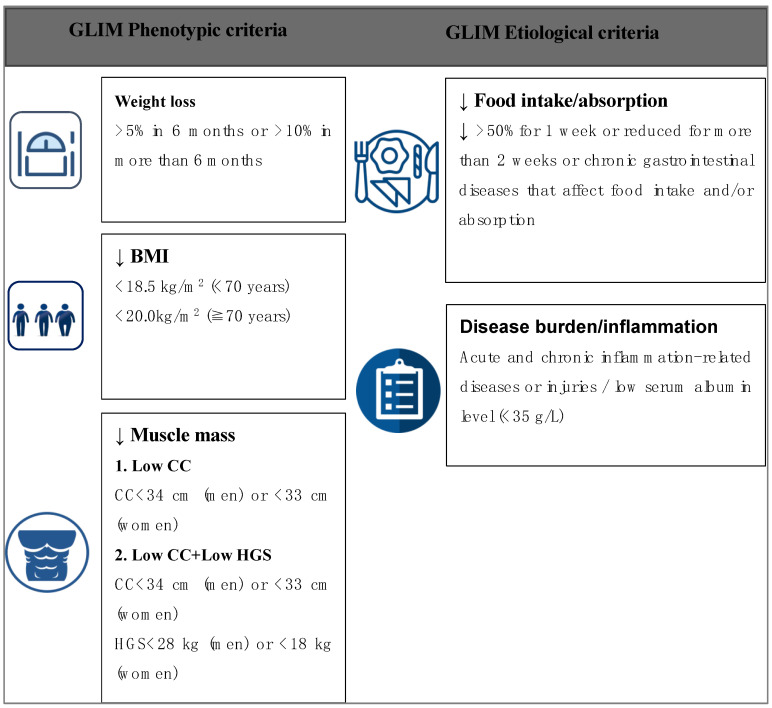
The different phenotypic and etiological criteria that were used in the 33 GLIM criteria combinations in the present study. GLIM, Global Leadership Initiative on Malnutrition; BMI, body mass index; CC, Calf circumference; HGS, Handgrip strength.

The implementation of the GLIM criteria [2] includes two steps. ① Screening for subjects with nutritional risks (with an NRS2002 score ≥ 3 points); ② Diagnosis of malnutrition according to the 33 sets of the GLIM criteria that use different combinations of the phenotypic and etiological criteria allowed by the GLIM committee (Figure 1). The phenotypic criteria include (1) Weight loss: unconscious weight loss of more than 5% in the past 6 months, or loss of more than 10% in more than 6 months. (2) BMI reduction: a BMI < 18.5 kg/m^2^ for people < 70 years or a BMI < 20 kg/m^2^ for people ≥ 70 years, as recommended by the GLIM committee for Asians [2]. (3) Muscle mass reduction: Two alternate criteria for muscle mass reduction were used in the current study because data on the body composition of the subjects are not available in the dataset. Criterion 1: reduction in the CC (CC < 34 cm in men or <33 cm in women); Criterion 2: CC reduction + HGS reduction (<28 kg in men or <18 kg in women). This practice is in line with previous studies and the consensus of the Asian working group for sarcopenia (AWGS) 2019 [9]. The etiological criteria include (1) reduced food intake or presence of digestive and absorption disorders: energy intake is reduced by more than 50% for more than one week, or energy intake is reduced for more than two weeks, or accompanied by chronic gastrointestinal diseases that affect food intake and/or absorption; (2) disease burden/inflammation: there are acute and chronic inflammation-related diseases or injuries, which are evaluated according to the subject’s history of acute and chronic diseases at admission. In this study, a decreased serum albumin level (<35 g/L) was used as an indicator of inflammation [19,20].

### 2.5. Statistical Analysis

The IBM SPSS statistical software (V26.0) was used to analyze the data. The normality of variables was evaluated with the Shapiro–Wilk test. Continuous variables conforming to the normal distribution were expressed as mean ± standard deviation (SD). Student’s *t*-test was used to compare the means of two independent groups. Continuous variables that did not conform to the normal distribution were expressed as the median and the quartile deviation (QD), and the Mann–Whitney U test was used to compare the differences between two independent samples. The consistencies of the malnutrition diagnosis between the different combinations of the GLIM criteria and the SGA scale were evaluated according to the Kappa statistics. A Kappa score between 0 and 0.20 indicates weak consistency; 0.20–0.4 indicates low consistency; 0.4–0.6 indicates medium consistency; 0.6–0.8 indicates good consistency; 0.8–1 indicates excellent consistency. Spearman’s rank correlation was used to analyze the covariates of the exposure variables. Logistic regression was used to assess the associations between malnutrition diagnosed with the different GLIM combinatory criteria and the incidences of IHC and PLOS. The contribution of each exposure variable to the outcome variables was evaluated according to its odds ratio (OR). All statistical tests were bilateral and a *p*-value < 0.05 was considered statistically significant.

### 2.6. Machine Learning Algorithm

The R software (V3.6.3) was used to calculate the mean decrease in accuracy (MDA) for the different GLIM criteria combinations using the random forest algorithm. To generate the machine learning models, the dataset was split into 75% for training and 25% for testing. The model training process was repeated 500 times and the average performance metrics were determined. Each training run produces an individual decision tree. All decision trees form a random forest. MDA is the degree of decrease in the accuracy of random forest prediction after changing the value of a variable into a random number. The higher value indicates the higher relative importance of the variable in the model.

## 3. Results

### 3.1. Baseline Characteristics of the Subjects

**Figure 2:**
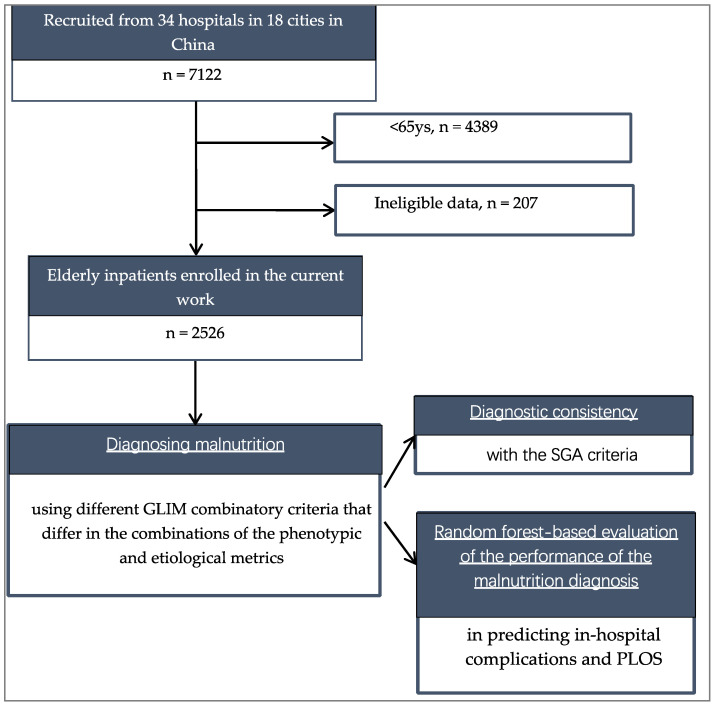
Flow chart of the study. GLIM, Global Leadership Initiative on Malnutrition; BMI, body mass index; CC, Calf circumference; HGS, Handgrip strength.

**Figure 3:**
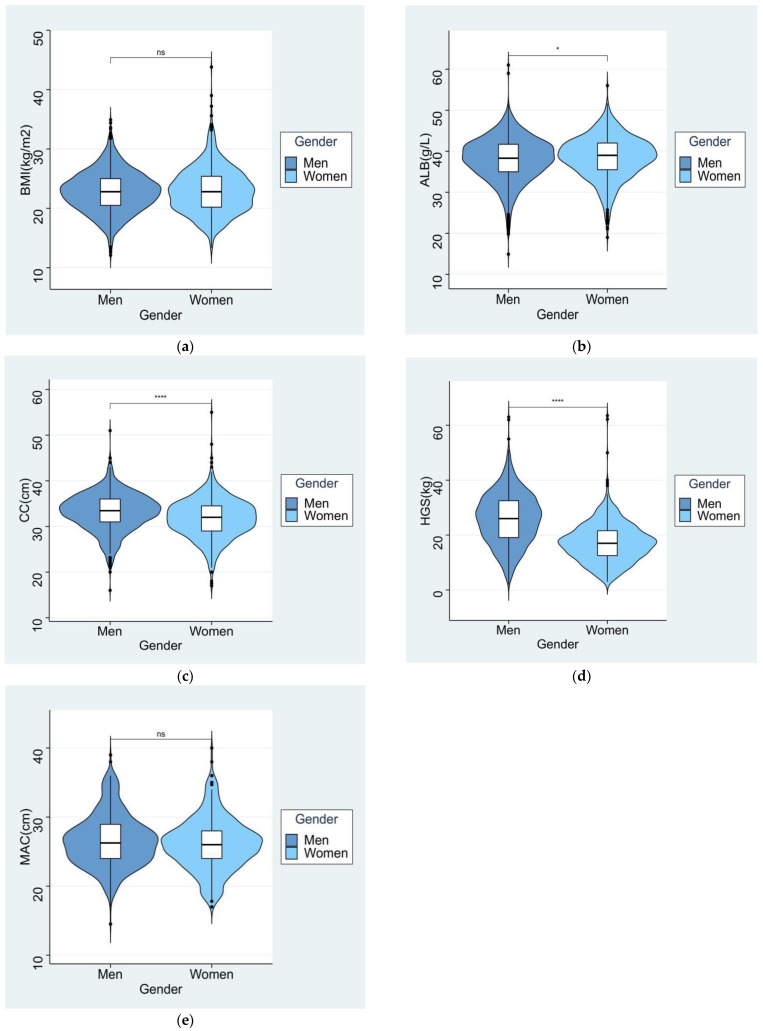
Differences in the level of the single diagnostic metrics between male and female subjects at admission. (**a**) Body mass index (BMI). (**b**) Albumin (ALB). (**c**) Calf circumference (CC). (**d**) Hand grip strength (HGS). (**e**) Mid-upper arm circumference (MAC). **** *p* < 0.001, * *p* < 0.05. ns, No significance.

After screening the registration data of 7122 hospitalized patients, we excluded 4389 patients who were <65 years of age at enrollment and 207 patients whose data were ineligible according to the inclusion/exclusion criteria. As a consequence, we included 2526 subjects in the current study (Figure 2). Table 1 summarizes the baseline characteristics of the subjects at admission. The average age of the participants was 74.63 ± 7.12 years and the age of the subjects was not significantly different between males and females. The proportion of male patients (59.2%) was higher than that of females. The proportions of married males were not significantly different from females (*p* > 0.05). The BMI of males was not significantly different from females (*p* > 0.05, Figure 3a), but the height, weight, and education level were significantly higher in men than in women. The leading reasons for hospitalization were tumors (37.5%), digestive system diseases (18.3%), and nervous system diseases (17.0%). The proportions of people with endocrine, nervous, respiratory, cardiovascular, and kidney diseases in men were not significantly different from women (*p* > 0.05), but the prevalence of tumors was significantly higher in men than in women; The prevalence of bone joint and digestive system diseases was significantly higher in women than in men (*p* < 0.05).

Regarding blood parameters, except for the serum hemoglobin concentration, which was significantly higher in men than in women, all the other parameters of female patients, including serum total protein, albumin (Figure 3b), triglyceride, and total cholesterol, were significantly higher than males (*p* < 0.05). Blood lymphocyte count was also significantly higher in women than in men (*p* < 0.05).

A total of 829 (32.8%) subjects were diagnosed with malnutrition at admission according to the SGA criteria, and the SGA score was not significantly different between men and women (*p* > 0.05). As expected, the CC (Figure 3c) and HGS (Figure 3d) were higher in men than in women (*p* < 0.05), and there was no difference in the MAC (Figure 3e) between genders.

### 3.2. Prevalence of the Single GLIM Phenotypic and Etiological Criteria in the Subjects

The prevalence of the individual phenotypic and etiological criteria used in the combinatory GLIM criteria are shown in Table 2. A reduced CC was seen in 49.1% of the subjects, while a reduced BMI was seen in only 17.0% of the subjects, representing the most and the least prevalent phenotypic criteria, respectively. The incidence of CC reduction was higher in women than in men (*p* < 0.05). The prevalence of weight loss and disease burden/inflammation was higher in men than in women. There were no significant differences in BMI reduction, CC reduction, HGS reduction, and low food intake/absorption between male and female inpatients (*p* > 0.05).

### 3.3. Prevalence of Malnutrition Diagnosed with the 33 GLIM Criteria Combinations and Their Diagnostic Consistency with the SGA Criteria

As shown in Table 3, the malnutrition rates detected with the 33 combinatory GLIM criteria range from 3.3% (GLIM 33, WBM_2_+FD) to 27.2% (GLIM 5, M_1_+F). Compared to the SGA criteria, the Kappa agreement coefficients of these combinations ranged from 0.130 to 0.866, among which GLIM 1 (W+F), GLIM 2 (W+D), GLIM 3 (B+F), GLIM 4 (B+D), GLIM 9 (WF+D), GLIM 12 (M_2_+FD), and GLIM 24 (WM_1_+FD) showed a moderate agreement with the SGA criteria, accounting for 21.2% of the combinations; GLIM 6 (M_2_+F), GLIM 8 (M_2_+D), and GLIM 11 (M_1_+FD) showed a good agreement with the SGA criteria, accounting for 9.1% of the combinations; GLIM 5 (M_1_+F) and GLIM 7 (M_1_+D) showed an excellent agreement with the SGA criteria, accounting for 6.1% of the combinations.

### 3.4. Associations of Malnutrition Diagnosed with the 33 GLIM Criteria Combinations with the Total 30-Day IHC and PLOS in Elderly Patients

A total of 103 (4.1%) subjects were inflicted with various complications within 30 days of hospitalization, including 62 infectious complications (2.5%) and 41 non-infectious complications (1.6%). Spearman’s rank correlation coefficients in Supplementary Table S1 reveal that age, gender, and marital status are independent exposure variables among all demographic, anthropometric, and hematological parameters, and reasons for hospitalization. Thus, to investigate the relationships between the malnutrition diagnosis made by the 33 GLIM criteria combinations and the incidence of the 30-day IHC of the subjects, the presence or absence of malnutrition was used as an exposure variable along with age, gender, and marital status as covariates and analyzed using logistic regression. Marital status was included in the model because it can influence the emotions and food intake of the subjects and is closely related to the nutritional status of the subjects. Table 4 shows that, except for GLIM 6 (M_2_+F) and GLIM 31 (WBM_2_+D), all the other combinations were significantly positively associated with the presence of IHC in the subjects (*p* < 0.05), including those with low or moderate diagnostic consistency with the SGA criteria (GLIM 15, GLIM 17, GLIM 24, and GLIM 32). The ORs of GLIM 15 (WM_1_+D), GLIM 17 (WM_1_+F), GLIM 24 (WM_1_+FD), and GLIM 32 (WBM1+FD) were as high as 3.336 (95% CI, 2.100–5.299), 3.074 (95% CI, 1.915–4.935), 3.557 (95% CI, 2.267–5.582), and 3.082 (95% CI, 1.718–5.530), respectively, suggesting that these GLIM criteria combinations are useful for predicting.

The mean length of hospital stay was 14.24 ± 6.25 days. A total of 989 subjects were influenced by PLOS, accounting for 39.2% of the subjects. To investigate the relationships between the malnutrition diagnosis made using the 33 GLIM criteria combinations and the incidence of PLOS in elderly inpatients, we took PLOS as the outcome variable, malnutrition as an exposure variable along with age, gender, and marital status as covariates in logistic regression models. The ORs in Table 4 show that, except for GLIM 1, GLIM 2, GLIM 6, GLIM 7, GLIM 8, GLIM 9, GLIM 11, GLIM 12, GLIM 16, GLIM 18, and GLIM 25 (*p* > 0.05), the remaining combinations are all significantly associated with PLOS in the subjects (*p* < 0.05).

### 3.5. Performance of Malnutrition Defined by Different GLIM Criteria Combinations in Predicting Total IHC and PLOS in Elderly Inpatients

**Figure 4:**
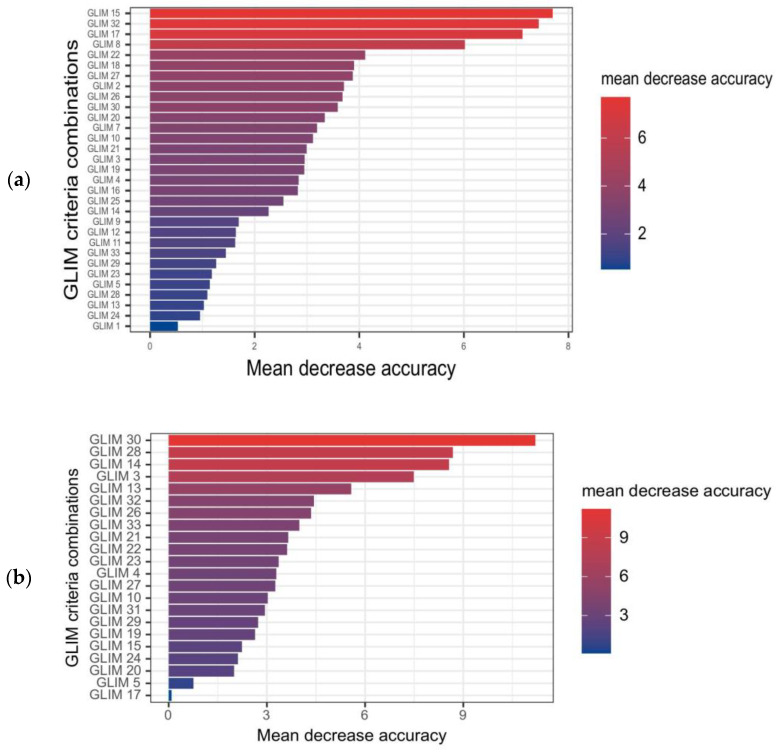
The rank of the MDAs of the GLIM criteria combinations in predicting adverse hospitalization outcomes in elderly patients as revealed by the random forest models. (**a**) MDAs in predicting IHC. (**b**) MDAs in predicting PLOS. MDA, mean decrease accuracy; GLIM, Global Leadership Initiative on Malnutrition; PLOS, prolonged length of hospital stay.

To increase the prediction accuracy, we further evaluated the performance of the GLIM criteria combinations that showed a significant association with IHC and PLOS in elderly inpatients with random forest models. Age, gender, and marital status were included in the models as covariates. The MDAs in Figure 4a show that GLIM 15 (WM1+D), GLIM 32 (WBM_1_+FD), GLIM 17 (WM_1_+F), and GLIM 8 (M_2_+D) performed better than the other combinations in predicting IHC because their MDAs (7.698, 7.431, 7.121, and 6.019, respectively) rank higher than all the other combinations. Figure 4b shows that the MDA of GLIM 30 (WBM_1_+D) (11.202) is the highest in all of the GLIM combinations in predicting PLOS, followed by GLIM 28 (WBM_1_+F), GLIM14 (WB+D), and GLIM 3(B+F).

## 4. Discussion

### 4.1. Principal Findings

In this study, the results of the random forest model indicate that GLIM 15 (unconscious weight loss + muscle mass reduction identified by a reduced CC, combined with disease burden/inflammation) performs best in predicting the total 30-day IHC in elderly patients, while GLIM 30 (unconscious weight loss, muscle mass reduction identified by a reduced CC and BMI reduction, combined with disease burden/inflammation) performs best in predicting PLOS in elderly inpatients. CC reduction is the most prevalent single phenotypic criterion that affects nearly half (49.1%) of elderly inpatients, and the reduction is more prevalent in females than males (Table 2). The GLIM criteria combinations that use CC as the main measure for muscle mass reduction perform consistently better than those using CC reduction + HGS reduction.

Reduced muscle mass is one of the key phenotypic criteria under the GLIM framework [7]. However, the metric or metric combinations that best reflect muscle loss vary depending on the specific clinical settings where they are used. Our findings are in agreement with a previous study conducted on emergency geriatric inpatients which found that diagnosing malnutrition by assessing muscle mass reduction through CC was independently associated with adverse outcomes (transference to intensive care unit and in-hospital mortality) [21]. Sanchez Rodriguez et al. also found that, in elderly community-dwellers, the GLIM criteria combinations that use CC as the metric for muscle mass reduction were the best at predicting mortality, superior to HGS and MAC [22].

Our findings are also in line with the report that CC is highly correlated with the direct measurements of skeletal muscle mass [23] and can capture aging-related muscle mass loss [24]. CC is also the most commonly used tool to evaluate skeletal muscle mass in the diagnosis of sarcopenia [25].

As to other anthropometric parameters, CC reduction + HGS reduction performs worse than CC reduction alone at predicting 30-day IHC and PLOS in elderly patients, although the function of skeletal muscle is considered to be correlated with the mass of the muscle. It is believed that, in some circumstances, the functional decline of the muscle may precede the loss of muscle mass, so HGS and CC + HGS have also been suggested as substitutes for muscle mass reduction under the GLIM criteria. Contreras-Bolivar et al. have shown that using HGS as a GLIM phenotypic criterion for diagnosing malnutrition can predict the 6-month mortality of cancer patients [26]. In contrast, many studies have reported that muscle function is not enough to replace muscle mass and the changes in muscle function may be inconsistent with the changes in muscle mass, particularly in a disease state [7]. Zhang et al. found that the decline in HGS was only a weak predictor of CT-detected muscle mass reduction in patients with gastric cancer [27]. Yin et al. found that, compared with CC+HGS, CC alone appears to be equal in terms of its ability to evaluate muscle mass decreases under the GLIM framework [28]. Moreover, if HGS was included in the GLIM criteria in addition to a reduced CC as a combinatory indicator for muscle loss in people with diseases, they may underestimate the rate of malnutrition and adversely affect the prediction of prognosis. Overall, our results, along with these previous studies, provide evidence that CC is a pragmatic measure for muscle loss in many clinical situations.

In the present study, only 17.0% of subjects had a decreased BMI, whereas the prevalence of other single phenotypic and etiological manifestations of malnutrition were high. This is consistent with a multitude of reports that the proportion of overweight and obesity in patients with various diseases has been increasing in recent years and that BMI is unable to distinguish between the lean and fat mass of the body, restricting its value in identifying people with sarcopenia and malnutrition.

In the present study, MAC reduction was not used in the GLIM criteria combinations. Because there is not a widely accepted cut-off value for MAC reduction, Henrique et al. have used a self-defined cut-off value to evaluate MAC reduction and used CC, MAC, and fat-free body mass measured using DXA to evaluate muscle mass reduction in their 10 GLIM criteria combinations that contain only one phenotypic and one etiological criterion [6]. They found that random forest is the most effective machine learning model for predicting postoperative complications with the 10 combinations in gastrointestinal surgery patients, and that MAC alone, as well as fat-free body mass+inflammation, were the most relevant GLIM criteria for predicting surgical complications. The average age (58.5 years) of their study participants was younger than ours, with only 45.9% being over 60 years old, and 53.4% of them being women. Our criteria combinations are more complex than theirs. Nevertheless, we believe that the value of MAC in evaluating muscle mass loss needs to be further established if the optimal threshold for MAC reduction in the Chinese elderly can be determined in the future.

In the current work, the prevalence of unconscious weight loss and disease burden/inflammation is higher in men than in women (Table 2), which may be related to the high proportion of cancer patients in men (Table 1). This may also be the reason why the concentrations of serum albumin, triglyceride, total cholesterol concentrations, and lymphocyte count were lower in male than in female patients (Table 1). As to the two GLIM etiological criteria, we find that disease burden/inflammation was present in 44.1% of the patients and is the most suitable etiological GLIM criterion for predicting adverse prognosis in the current study population, although low food intake/absorption also existed in 48.3% of the patients (Table 2).

In this work, we found that malnutrition diagnoses made using most of the GLIM criteria combinations were related to adverse clinical outcomes (total IHC and PLOS) although the diagnostic consistencies of these GLIM combinations with the SGA criteria varied widely. This is consistent with a previous study [29] on the Chinese elderly which found that the GLIM criteria were superior to the SGA criteria in predicting postoperative complications. Nicole et al. have evaluated the performance of 21 GLIM criteria combinations in predicting the mortality of tumor patients (49.0% were ≥65 years old) and found that the combination of weight loss and muscle mass reduction with any one of the GLIM etiological criteria are the most important GLIM criteria combinations to predict the mortality of tumor patients [4]. Their study population, muscle mass assessment methods, criteria combinations, sample size, and main findings are similar to ours.

This study is the first to explore the value of different GLIM phenotypic and etiological criteria combinations in predicting 30-day IHC and PLOS in an elderly Chinese population with various diseases, in which anthropometric parameters were used as the main metrics for muscle mass reduction assessment. This study is representative of elderly inpatients in China because it is based on a nationwide multicenter cohort study with large sample size, covering a variety of diseases of elderly inpatients. The results are useful for predicting the prognosis for this population in the future. Moreover, our results are relevant in that the metrics used to evaluate muscle mass reduction in the present study, namely CC and HGS, are simple and pragmatic for many clinical settings where expensive instruments and experienced clinicians are lacking.

### 4.2. Limitations

This study has limitations. First, because this study is a retrospective study, the muscle mass of the participants had not been assessed with a gold-standard method (DXA, MRI, etc.) so we cannot compare the results of CC with it. Second, the cut-off value for CC reduction in different populations is still controversial in some regions of the world, so the value of CC in diagnosing malnutrition and predicting prognosis needs to be further established in more clinical settings. In the future, more well-designed prospective studies that determine the muscle mass of the subjects with a gold standard are needed to further substantiate the performance of CC in diagnosing malnutrition under the GLIM criteria. In addition, the optimal cut-off value for CC reduction needs to be explored in different ages, genders, and ethnic groups, as well as pathophysiological backgrounds. Moreover, the applicability of other pragmatic anthropometric parameters (e.g., MAC) in these applications also needs to be further established.

## 5. Conclusions

In this study, most of the malnutrition diagnoses made using the 33 GLIM criteria combinations were well associated with 30-day IHC and PLOS in elderly Asian inpatients. Muscle mass reduction defined by a reduced CC performed well in the GLIM criteria combinations for diagnosing malnutrition and predicting IHC and PLOS in elderly Asian inpatients. The applicability of other anthropometric parameters in these applications needs to be further explored.

## Figures and Tables

**Table 1:** Baseline characteristics of the subjects at admission (n = 2526).

Characteristics	Men	Women	Total	*p*
n	1495(59.18)	1031(40.82)	2526	0.000
General characteristics				
Age (year)	74.73 ± 7.31	74.49 ± 6.82	74.63 ± 7.12	0.383
Married (%)	1401(93.7)	976(94.7)	2377(94.2)	0.318
Height * (cm)	168.91 ± 6.09	157.47 ± 6.00	164.24 ± 8.26	0.000
Weight * (kg)	65.11 ± 10.95	57.06 ± 10.43	61.83 ± 11.45	0.000
BMI (kg/m^2^)	22.78 ± 3.40	22.98 ± 3.81	22.86 ± 3.57	0.179
Education level *				
Primary school and lower	1013(67.8)	800(77.6)	1813(71.8)	0.000
High school	220(14.7)	111(10.8)	331(13.1)	
Bachelor’s degree or above	262(17.5)	120(11.6)	382(15.1)	
Hospitalization reasons				
Endocrine diseases	33(2.2)	30(2.9)	63(2.5)	0.266
Nervous system diseases	249(16.7)	181(17.6)	430(17.0)	0.554
Osteoarthropathy *	49(3.3)	92(8.9)	141(5.6)	0.000
Digestive diseases *	238(15.9)	225(21.8)	463(18.3)	0.001
Respiratory diseases	143(9.6)	80(7.8)	223(8.8)	0.116
Cardiovascular diseases	80(5.4)	58(5.6)	138(5.5)	0.765
Tumors *	615(41.1)	333(32.3)	948(37.5)	0.000
Kidney diseases	6(0.4)	4(0.4)	10(0.4)	0.958
Nutritional status according to the SGA criteria				
A(%)	983(65.8)	714(69.3)	1697(67.2)	0.180
B(%)	437(29.2)	269(26.1)	706(27.9)	
C(%)	75(5.0)	48(4.7)	123(4.9)	
Blood parameters				
Total protein * (g/L)	65.04 ± 6.90	66.06 ± 6.94	65.45 ± 6.93	0.001
Triglyceride * (mmol/L)	1.17(0.46)	1.36(0.61)	1.24(0.50)	0.000
Total cholesterol * (mmol/L)	3.96 ± 1.40	4.28 ± 1.53	4.08 ± 1.46	0.000
Hemoglobin * (g/L)	125.64 ± 21.34	117.81 ± 18.10	122.43 ± 20.44	0.000
Lymphocytes * (10^9^/L)	1.45(0.53)	1.58(0.55)	1.57(0.59)	0.001

* *p* < 0.05: between men and women; BMI: body mass index; SGA: subjective global assessment.

**Table 2:** Prevalence of the single GLIM phenotypic and etiological criteria in the subjects.

	Men	Women	Total	*p*
Phenotypic criteria				
Weight loss *	349(23.3)	203(19.7)	552(21.9)	0.029
BMI reduction	243(16.3)	187(18.1)	430(17.0)	0.216
CC reduction *	691(46.2)	550(53.3)	1241(49.1)	0.000
CC reduction+HGS reduction	483(32.3)	335(32.5)	818(32.4)	0.922
Etiological criteria				
Low food intake or absorption	718(48.0)	503(48.8)	1221(48.3)	0.707
Disease burden or inflammation *	687(46.0)	428(41.5)	1115(44.1)	0.027

* *p* < 0.05 between men and women. BMI, body mass index; GLIM, Global Leadership Initiative on Malnutrition; CC, Calf circumference; HGS, Handgrip strength.

**Table 3:** Prevalence of malnutrition detected with the 33 GLIM criteria combinations and their diagnostic consistencies with the SGA criteria.

GLIM Criteria Combinations	Malnourished Patients, n (%)	Kappa vs. SGA	95% CI
Combinations with 1 phenotypic and 1 etiological criterion			
GLIM 1 (W+F)	409 (16.2)	0.567	(0.532–0.602)
GLIM 2 (W+D)	408 (16.2)	0.581	(0.546–0.616)
GLIM 3 (B+F)	269 (10.6)	0.566	(0.531–0.601)
GLIM 4 (B+D)	319 (12.6)	0.429	(0.397–0.460)
GLIM 5 (M1+F)	686 (27.2)	0.866	(0.827–0.904)
GLIM 6 (M2+F)	479 (19.0)	0.648	(0.611–0.684)
GLIM 7 (M1+D)	653 (25.9)	0.833	(0.795–0.871)
GLIM 8 (M2+D)	453 (17.9)	0.618	(0.582–0.654)
Combinations with 2 phenotypic and 1 etiological criterion			
GLIM 9 (WF+D)	327 (12.9)	0.467	(0.434–0.500)
GLIM 10 (BF+D)	219 (8.7)	0.325	(0.297–0.354)
GLIM 11 (M1+FD)	445 (17.6)	0.609	(0.573–0.645)
GLIM 12 (M2+FD)	315 (12.5)	0.452	(0.419–0.484)
GLIM 13 (WB+F)	142 (5.6)	0.217	(0.193–0.242)
GLIM 14 (WB+D)	171 (6.8)	0.259	(0.233–0.285)
GLIM 15 (WM1+D)	264 (10.5)	0.386	(0.355–0.417)
GLIM 16 (WM2+D)	197 (7.8)	0.341	(0.311–0.371)
GLIM 17 (WM1+F)	257 (10.2)	0.376	(0.346–0.407)
GLIM 18 (WM2+F)	186 (7.4)	0.280	(0.253–0.307)
GLIM 19 (BM1+F)	226 (8.9)	0.335	(0.306–0.364)
GLIM 20 (BM2+F)	172 (6.8)	0.260	(0.234–0.287)
GLIM 21 (BM1+D)	265 (10.5)	0.396	(0.365–0.428)
GLIM 22 (BM2+D)	199 (7.9)	0.298	(0.270–0.326)
Combinations with 2 phenotypic and 2 etiological criteria			
GLIM 23 (WB+FD)	128 (5.1)	0.197	(0.174–0.220)
GLIM 24 (WM1+FD)	287 (11.4)	0.416	(0.384–0.447)
GLIM 25 (WM2+FD)	166 (6.6)	0.252	(0.226–0.278)
GLIM 26 (BM1+FD)	182 (7.2)	0.274	(0.248–0.301)
GLIM 27 (BM2+FD)	141 (5.6)	0.216	(0.192–0.240)
Combinations with 3 phenotypic and 1 etiological criterion			
GLIM 28 (WBM1+F)	121 (4.8)	0.187	(0.164–0.209)
GLIM 29 (WBM2+F)	91 (3.6)	0.142	(0.122–0.162)
GLIM 30 (WBM1+D)	130 (5.1)	0.200	(0.177–0.223)
GLIM 31 (WBM2+D)	99 (3.9)	0.154	(0.133–0.175)
Combinations with 3 phenotypic and 2 etiological criteria			
GLIM 32 (WBM1+FD)	142 (5.6)	0.217	(0.193–0.242)
GLIM 33 (WBM2+FD)	83 (3.3)	0.130	(0.111–0.149)

Notes: GLIM, Global Leadership Initiative on Malnutrition; W, Weight loss; B, body mass index reduction; M_1_, calf circumference (CC) reduction; M_2_, CC reduction+HGS reduction; F, Food intake reduction; D, Disease burden/inflammation.

**Table 4:** Associations of malnutrition defined by the 33 GLIM criteria combinations with the incidence of IHC and PLOS in elderly inpatients.

GLIM Criteria Combinations	In-Hospital Complications				PLOS	
	OR	95% CI	*p*	OR	95% CI	*p*
GLIM 1	2.220	1.428–3.450	0.000	1.199	0.966–1.489	0.100
GLIM 2	2.579	1.676–3.969	0.000	1.241	0.999–1.541	0.051
GLIM 3	2.155	1.290–3.602	0.003	1.354	1.046–1.752	0.021
GLIM 4	2.074	1.275–3.375	0.003	1.288	1.013–1.637	0.039
GLIM 5	2.076	1.351–3.040	0.001	1.217	1.016–1.458	0.033
GLIM 6	1.537	0.972–2.431	0.066	1.118	0.962–1.450	0.112
GLIM 7	2.710	1.806–4.065	0.000	1.182	0.983–1.421	0.075
GLIM 8	1.849	1.176–2.908	0.008	1.094	0.885–1.351	0.407
GLIM 9	2.827	1.804–4.432	0.000	1.219	0.962–1.546	0.101
GLIM 10	2.380	1.395–4.061	0.001	1.398	1.055–1.852	0.020
GLIM 11	2.849	1.869–4.344	0.000	1.234	1.000–1.521	0.050
GLIM 12	2.224	1.375–3.597	0.001	1.138	0.983–1.450	0.297
GLIM 13	2.369	1.259–4.458	0.007	1.864	1.321–2.630	0.000
GLIM 14	2.081	1.134–3.822	0.018	1.891	1.379–2.593	0.000
GLIM 15	3.336	2.100–5.299	0.000	1.364	1.053–1.767	0.019
GLIM 16	2.097	1.185–3.714	0.011	1.273	0.947–1.711	0.109
GLIM 17	3.074	1.915–4.935	0.000	1.376	1.059–1.787	0.017
GLIM 18	2.241	1.264–3.975	0.006	1.311	0.968–1.775	0.080
GLIM 19	2.668	1.589–4.481	0.000	1.520	1.151–2.008	0.003
GLIM 20	2.698	1.528–4.763	0.001	1.457	1.063–1.997	0.019
GLIM 21	2.473	1.502–4.072	0.000	1.480	1.142–1.918	0.003
GLIM 22	2.228	1.268–3.916	0.005	1.437	1.070–1.930	0.016
GLIM 23	2.676	1.419–5.047	0.002	1.851	1.290–2.656	0.001
GLIM 24	3.557	2.267–5.582	0.000	1.319	1.027–1.694	0.030
GLIM 25	2.372	1.314–4.280	0.004	1.086	0.786–1.502	0.617
GLIM 26	2.983	1.729–5.117	0.000	1.667	1.228–2.265	0.001
GLIM 27	2.839	1.560–5.169	0.001	1.605	1.137–2.266	0.007
GLIM 28	2.876	1.520–5.439	0.001	2.160	1.485–3.142	0.000
GLIM 29	2.378	1.114–5.075	0.025	2.104	1.370–3.229	0.001
GLIM 30	2.880	1.557–5.328	0.001	2.173	1.514–3.121	0.000
GLIM 31	2.120	0.997–4.511	0.051	1.999	1.328–3.011	0.001
GLIM 32	3.082	1.718–5.530	0.000	1.577	1.118–2.224	0.009
GLIM 33	2.647	1.237–5.665	0.012	2.073	1.326–3.241	0.000

Notes: GLIM, Global Leadership Initiative on Malnutrition; OR, odds ratio; CI, confidence interval; PLOS, prolonged length of hospital stay.

## Data Availability

Data will be available from the corresponding author upon reasonable request.

## Supplementary Materials

Supplementary data to this article can be found online at https://www.mdpi.com/article/10.3390/nu15194146/s1, Table S1: Spearman’s rank correlation between the exposure variables of the subjects.
