# Supplementary material for: Machine Learning-Based Prediction of Complications and Prolonged Hospitalization with the GLIM Criteria Combinations Containing Calf Circumference in Elderly Asian Patients
Source: Nutrients. 2023 Sep 26;15(19):4146. doi: 10.3390/nu15194146 (PMC10574169; doi:10.3390/nu15194146)
Supplement: Supplementary file 1 [file nutrients-15-04146-s001.zip › nutrients-2609153-SI.pdf]

Table S1. Spearman's rank correlation between the exposure variables of the subjects

	Age	Gender	Marriage	Education	Height	Weight	BMI	Grip strength	Mid-upper-arm circumference	Calf circumference	Lymphocytes	Hemoglobin	Total protein	Albumin	Prealbumin	Triglycerides	Total cholecsterol	Hospitalization reasons
Age	1.000	-0.007	0.013	0.101**	-0.065**	-0.098**	-0.077**	-0.293**	-0.087**	-0.116**	-0.046*	-0.107**	-0.152**	-0.173**	-0.150**	-0.133**	-0.131**	-0.124**
Gender	-0.007	1.000	-0.020	-0.108**	-0.697**	-0.354**	0.009	-0.465**	-0.053	-0.182**	0.066**	-0.218**	0.076**	0.041*	0.038	0.130**	0.144**	-0.071**
Marriage	0.013	-0.020	1.000	-0.024	0.024	0.020	0.018	0.019	0.049	0.029	0.055**	0.019	0.029	0.037	0.078*	0.008	0.007	-0.097**
Education	0.101**	-0.108**	-0.024	1.000	0.119**	0.107**	0.048*	.0108**	0.014	0.094**	-0.024	0.021	-0.030	0.043*	0.047	-0.023	-0.067**	-0.031
Height	-0.065**	-0.697**	0.024	0.119**	1.000	0.542**	0.020	0.449**	0.150**	0.285**	-0.016	0.188**	-0.039	-0.026	-0.005	0.048*	-0.140**	0.041*
Weight	-0.098**	-0.354**	0.020	0.107**	0.543**	1.000	0.826**	0.378**	0.597**	0.628**	0.101**	0.279**	0.085**	0.152**	0.103**	0.155**	-0.047*	-0.046*
BMI	-0.077**	0.009	0.018	0.048*	0.020	0.826**	1.000	0.170**	0.610**	0.565**	0.137**	0.220**	0.132**	0.207**	0.125**	0.215**	0.025	-0.086**
Grip strength	-0.293**	-0.465**	0.019	0.108**	0.449**	0.378**	0.170**	1.000	0.194**	0.350**	0.010	0.265**	0.074**	0.159**	0.045	0.026	-0.012	0.110**
Mid-upper-arm circumference	-0.087**	-0.053	0.049	0.014	0.150**	0.597**	0.610**	0.194**	1.000	0.388**	0.108**	0.210**	0.088**	0.152**	0.068	0.177**	0.014	-0.081**
Calf circumference	-0.116**	-0.182**	0.029	0.094**	0.285**	0.628**	0.565**	0.350**	0.388**	1.000	0.079**	0.243**	0.099**	0.168**	0.093**	-0.084**	-0.057*	-0.013
Blood lymphocytes	-0.046*	0.066**	0.055**	-0.024	-0.016	0.101**	0.137**	0.010	0.108**	0.079**	1.000	0.225**	0.228**	0.226**	0.215**	0.244**	-0.176**	-0.139**
Hemoglobin	-0.107**	-0.218**	0.019	0.021	-0.188**	0.279**	0.220**	0.265**	0.210**	0.243**	0.225**	1.000	0.304**	0.438**	0.223**	0.138**	0.151**	-0.064**
Total protein	-0.152**	0.076**	0.029	-0.030	-0.039	-0.085**	0.132**	0.074**	0.088**	0.099**	0.228**	0.304**	1.000	0.647**	0.200**	0.146**	0.294**	-0.004
Albumin	-0.173**	0.041*	0.037	0.043*	-0.026	0.152*	0.207**	0.159**	0.152**	0.168**	0.226**	0.438**	0.647**	1.000	0.282**	0.258**	0.258**	-0.059**
Prealbumin	-0.150**	0.038	0.078*	0.047	-0.005	0.103**	0.125**	0.045	0.068	-0.093**	0.215**	0.223**	0.200**	0.282**	1.000	0.220**	0.213**	-0.076*
Triglycerides	-0.133**	0.130**	0.008	-0.023	-0.048*	0.155**	0.215**	0.026	0.177**	0.084**	0.244**	0.138**	0.146**	0.258**	0.220**	1.000	0.077**	-0.109**

Total cholesterol	-0.131**	0.144**	0.007	-0.067**	-0.140**	-0.047*	0.025	-0.012	0.014	-0.057*	0.176**	0.151**	0.294**	0.258**	0.213**	0.077**	1.000	0.060*
Hospitalization reasons	-0.124**	-0.071**	-0.097**	-0.031	0.041*	-0.046*	-0.086**	0.110**	-0.081**	-0.013	-0.139**	-0.064**	-0.004	-0.059**	-0.076*	-0.109**	0.060*	1.000

*p<0.05, **P<0.01.
